# Peptide-Functionalized Silk Fibers as a Platform to Stabilize Gelatin for Use in Ingestible Devices

**DOI:** 10.3390/molecules27144605

**Published:** 2022-07-19

**Authors:** Luca Valentini, Lorenzo Pacini, Fosca Errante, Cecilia Morchio, Beatrice Sanna, Paolo Rovero, Antonino Morabito

**Affiliations:** 1 Civil and Environmental Engineering Department, University of Perugia, Strada di Pentima 4, 05100 Terni, Italy; 2 Interdepartmental Research Unit of Peptide and Protein Chemistry and Biology, Department of Chemistry “Ugo Schiff”, University of Florence, 59100 Sesto Fiorentino, Italy; l.pacini@unifi.it; 3 Interdepartmental Research Unit of Peptide and Protein Chemistry and Biology, Department of NeuroFarBa, University of Florence, 50019 Sesto Fiorentino, Italy; fosca.errante@unifi.it (F.E.); paolo.rovero@unifi.it (P.R.); 4 Dipartimento Neuroscienze, Psicologia, Area del Farmaco e della Salute del Bambino NEUROFARBA, Università degli Studi di Firenze, Viale Pieraccini 6, 50121 Firenze, Italy; cecilia.morchio@unifi.it (C.M.); beatrice.sanna@unifi.it (B.S.); antonino.morabito@unifi.it (A.M.)

**Keywords:** peptide, silk fibroin, programmable biomaterials, mechanical properties

## Abstract

The combination of pharmacologic and endoscopic therapies is the gold standard for treating intestinal failures. The possibility of chemical solubility in water is mandatory for intelligent capsules. Functionalised silk fibroin with peptides and covalently linking different molecular entities to its structure make this protein a platform for preparing gels dissolving in the small and large intestine for drug delivery. In the present study, we linked a peptide containing the cell-adhesive motif Arginine–Glycine–Aspartic acid (RGD) to degummed silk fibres (DSF). Regenerated silk fibroin (RS) films obtained by dissolving functionalised DSF in formic acid were used to prepare composite gelatin. We show that such composite gelatin remains stable and elastic in the simulated gastric fluid (SGF) but can dissolve in the small and large intestines’ neutral-pH simulated intestine fluid (SIF). These findings open up the possibility of designing microfabricated and physically programmable scaffolds that locally promote tissue regeneration, thanks to bio-enabled materials based on functionalised regenerated silk.

## 1. Introduction

The development of ingestible devices has been recently considered as a valid alternative to endoscopic and invasive clinical techniques for a broad range of applications, from ingestible electronics for physiological monitoring of a patient to programmable dosage of drugs [1,2,3,4,5,6,7,8,9].

These devices are often made of, at least in part, elastic polymers for packaging such devices [10]. The main drawback is that this approach relies on complications caused by the migration of fractured components that requires surgical solutions for their removal [11,12,13,14,15].

Gelatin is a collagen material that can be dissolved in water at 37 °C and is generally used for hydrogels in the fabrication of scaffolds for tissue engineering and drug delivery systems [16,17]. However, without crosslinking agents, it suffers from rapid dissolution in gastric and intestinal fluids [18,19]. Recently, inspired by silk fibroin due to its impressive biocompatibility, biodegradability, and mechanical properties [20], a new class of hydrogels has been brought to the attention of the scientific community, i.e., it was found that collagen interacted with silk fibroin in aqueous solution making possible the fabrication of scaffolds where drugs can be embedded and released at controlled rates [21,22]. A further advantage of this novel method to obtain hybrid collagens is the possibility of realising arginine–glycine–aspartic acid (RGD) functionalised silk fibroin nanoparticles to mitigate intestinal inflammatory state [23] as well as, for silk peptide, to improve energy and glucose metabolism and promote the gut microbiota’s homeostasis in obese young animals [24]. Incorporating exogenous RGD peptide on Bombyx mori silk fibroin membranes has been used in the ex vivo cultivation and transplantation of cells to treat severe eye diseases and reconstruct the ocular surface [25].

However, collagen is insoluble or at least is partially insoluble. Thus, the challenge is transforming water-soluble collagen into water-stable films for ingestible devices.

In the proposed method, the opportunity to covalently link synthetic peptides to degummed silk fibres (DSF) and then apply a reverse engineering approach to get regenerated silk fibroin (RS) films appears particularly relevant, offering the possibility of promoting the surface interactions between cells and functionalised biomaterials with designed architectural forms and structure [26,27,28]. For this purpose, several authors reported the use of cell-binding epitopes of the major adhesive proteins of the extracellular matrix (ECM), e.g., fibronectin, the well-known universal adhesive RGD peptide [23,29,30]. When grafted on nanomaterials, this peptide has been reported to enhance cell adhesion [23,29] or to enable specific cell targeting for drug delivery [30].

Accordingly, we synthesised a peptide containing the RGD motif (MO-07, sequence: YRGDS), endowed with anti-inflammatory and cell adhesion properties. We linked it to DSF by activating the -COOH groups on the backbone of DSF and subsequent functionalisation with the peptide. The synergistic effect of the functionalisation of DSF and the attachment of the peptide endowed the resultant regenerated silk film with remarkable differences in mechanical properties. The synergistic combination of MO-07/RS with gelatin stabilised the composite at 37 °C in a simulated gastric fluid (SGF) environment, showing that this compound remains elastic and stable in an acidic environment while partially dissolving in the small and large intestines’ simulated intestine fluid (SIF) environment.

## 2. Results and Discussion

**Figure 1:**
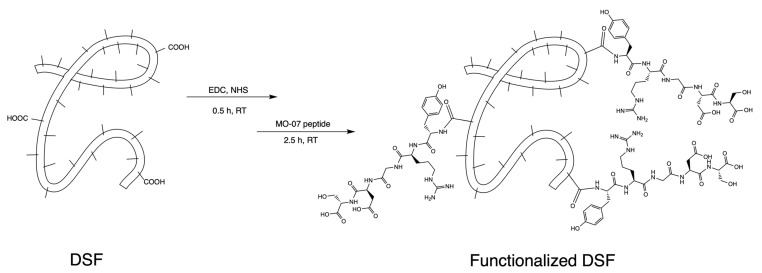
Scheme showing the synthetic pathway for activating -COOH residues on DSF and their reaction with the N-terminal amino group of MO-07 peptide.

Peptide MO-07 (structure in Figure S2) was covalently linked to carboxylic functions on the side chains of Asp and Glu residues of fibroin through its N-terminal amino group (Figure 1), according to previously reported methods with slight modifications [31]. This reaction required a pre-activation of the free -COOH groups belonging to aspartic acid or glutamic acid residues of fibroin that could be obtained using EDC/NHS as a coupling system. Quantitative chromatographic analysis of the reacted peptide indicated that 19–22% (n = 3) of the peptide was consumed by the reaction and therefore linked to the fibroin.

**Figure 2:**
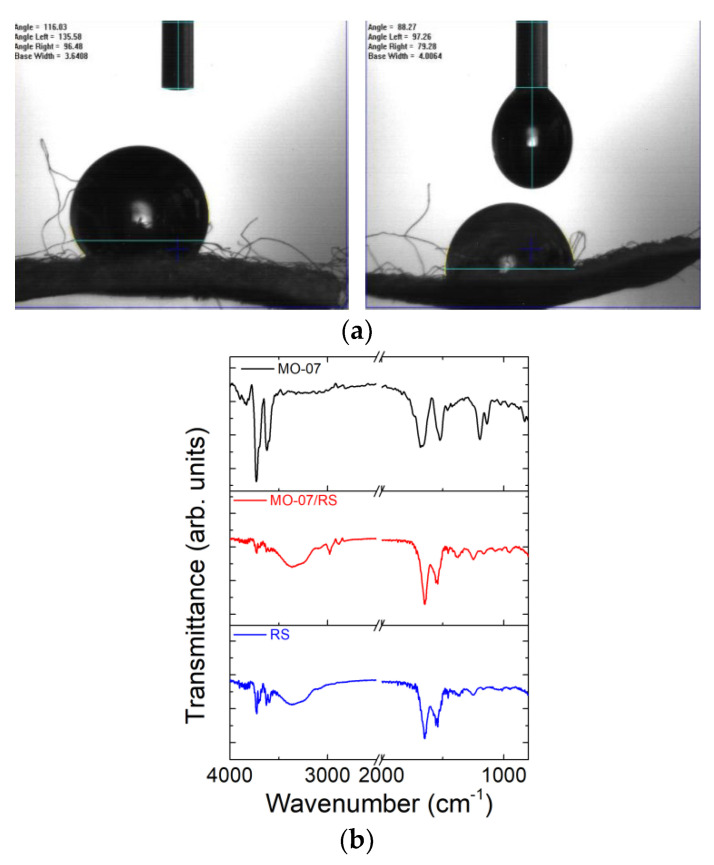
(**a**) Contact angle measurements of water on DSF (left panel) and MO-07/DSF (right panel). (**b**) FTIR spectra of MO-07 peptide, RS, and MO-07/RS films were obtained by dissolving DSF and peptide-functionalised DSF in formic acid, respectively.

The contact angles of water droplets on DSF and MO-07-modified DSF (Figure 2a) indicate that the DSF treated with MO-07 peptide exhibited lower contact angles (i.e., 88°) compared to those of neat DSF (i.e., 116°), indicating that the peptide formed a more hydrophilic surface on the silk fibre.

We proceeded with FTIR analysis to verify if the peptide was physisorbed or covalently linked to the silk fibres (Figure 2b). As-prepared RS film showed a strong amide I absorption band between 1652 cm^−1^ and 1647 cm^−1^ (random coils) and an amide II absorption band at 1541 cm^−1^ [32,33]. These peaks indicate a higher reduction in the crystalline secondary structure due to CaCl_2_ [34]. The new peaks at 2974 cm^−1^ and 2881 cm^−1^ in the spectrum of MO-07/RS (Figure 2b) belonged to the methylene C–H asymmetric/symmetric stretches of the MO-07 peptide. The peak at 3620 cm^−1^ can be attributed to the –OH stretch [35]. According to the obtained spectra, it is possible to observe a shift in the amide I band (from 1654 to 1637 cm^−1^); this could suggest amidation between the RS and MO-07 peptide by the grafting method [36,37].

**Figure 3:**
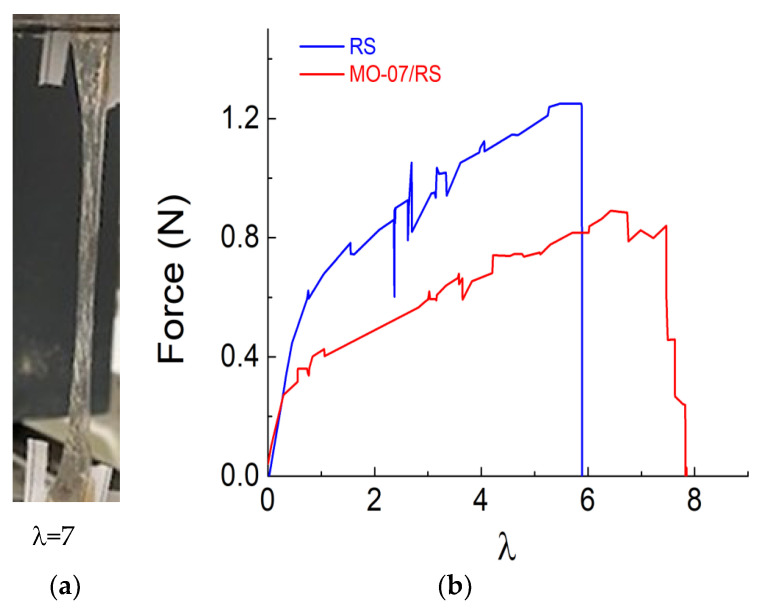
(**a**) Photograph demonstrating the elongation of MO-07/RS film when stretched and (**b**) force as a function of strain ratio (λ) curves for the RS and MO-07/RS samples, respectively.

The mechanical properties were evaluated in detail by assessing the practicability of the peptide-modified RS film as stretchable materials (Figure 3). The functionalisation effect of the peptide addition to the RS on the stretchability is illustrated in Figure 3a. As a result, the stretching properties, measured in terms of strain ratio λ = l/l_0_, where l_0_ is the initial length of the sample, of the MO-07/RS sample, were improved with higher strain at break. Compared with neat RS, we observed an increase in the elongation at break (Figure 3b). These mechanical properties can be explained as follows: the molecules of peptide hinder the secondary bonding between the protein chains (intra- and inter-molecular), establishing a mechanism reported elsewhere [38]. This new arrangement facilitates the sliding of the molecules so that the strength reduces while the elongation increases.

**Figure 4:**
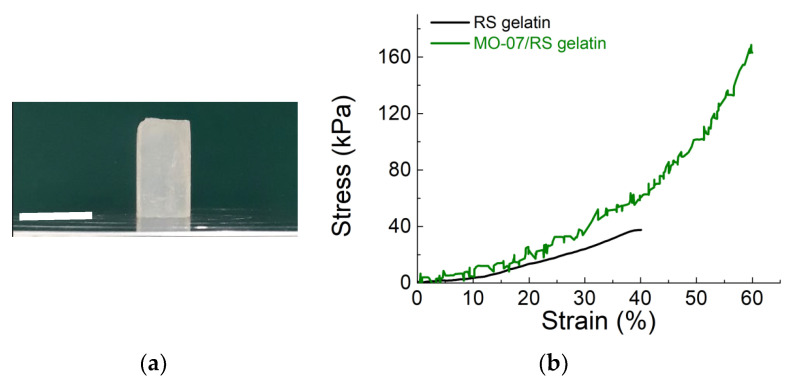
Compressive tests of the RS gelatin and MO-07/RS gelatin composite. (**a**) The MO-07/RS gelatin composite. The scale bar indicates 16 mm. (**b**) Stress-strain curves of RS gelatin and MO-07/RS gelatin composite.

Our previous study [34] reports a water-based method to produce RS composite gels. Briefly, the high content of Ca^2+^ ions dispersed in RS film allows the re-dispersion in water to obtain a gel. Combining the above points will enable us to adopt a solvent-free method for developing RS gelatin composites. We then tested the compressive strength of the RS gelatin and MO-07/RS composites (Figure 4). As depicted in Figure 4a, the MO-07/RS gelatin composite shows milky white and opaque colour. During the compressive test (compressed to 60%) of the RS gelatin and MO-07/RS gelatin composite (Figure 4b and Figure S1), it was clear that the RS gelatin shows linear elasticity, while the MO-07/RS sample did not exhibit any linear plateau region. From the comparisons of the corresponding stress-strain curves in Figure 4b, the composite gelatin achieved a high compression stress of 160 ± 8 kPa, which was about four times higher than that of RS gelatin gel (37 ± 7 kPa). These findings suggest gelatin’s mechanical properties can be tuned by adding peptide-modified RS.

**Figure 5:**
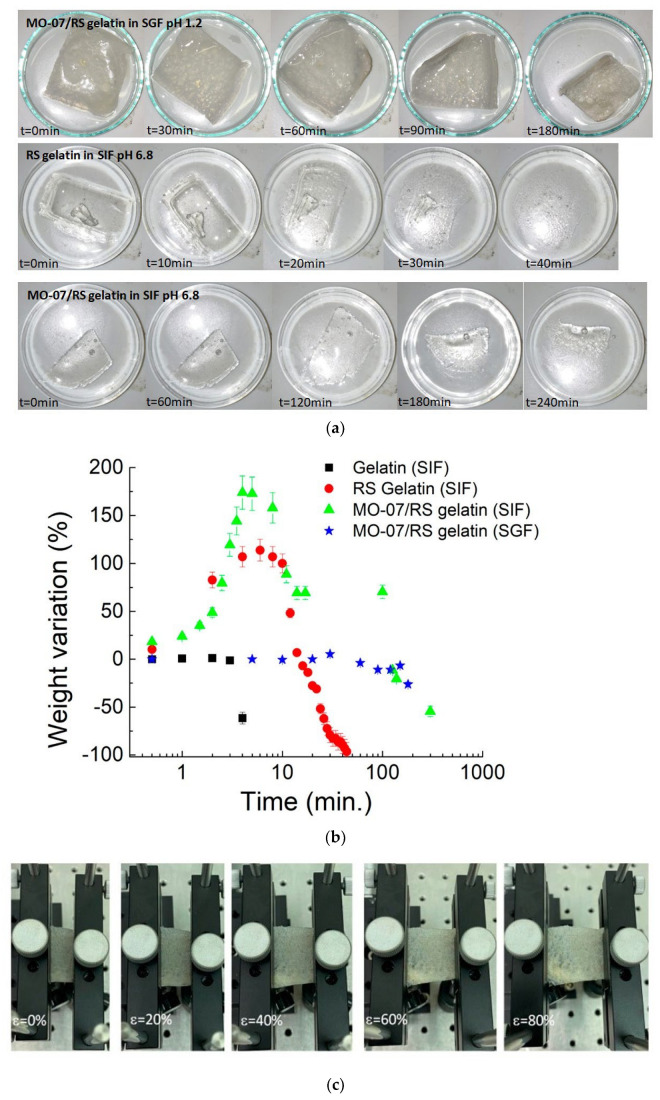
(**a**) Photographs show the consistency of degradation properties of RS gelatin and MO-07/RS gelatin composites in SIF and SGF. (**b**) Dissolution study of gelatin, RS gelatin, and MO-07/RS gelatin composites in simulated gastric fluid and simulated intestinal fluid showing the complete and partial dissolution of the RS gelatin and MO-07/RS gelatin composite in SIF, respectively, while no significant mass loss for the MO-07/RS gelatin composite in SGF. The vertical error bars correspond to the standard deviations of five samples per formulation. (**c**) Photographs of MO-07/RS gelatin composite at different strains after SGF treatment at 37 °C.

Similarly, the dissolution time has to be correlated to the mechanical properties. For our experiments, we consider the transit time as the time that the food takes to reach the large and small intestine, and it was estimated that the gastric time was between 35.5 min and 57 min, while the transit time for the small intestine was about 261 min [39,40]. The pH-dependent dissolution appearance and data of samples are reported in Figure 5a,b. Adding MO-07/RS to gelatin showed a stable degradation pattern at a pH value of 1.2. In contrast, for neutral pH values (i.e., 6.8), we observed a degradation pattern that indicated a total dissolution for the gelatin added with RS after 44 min and a weight loss of about 50% for the MO-07/RS-modified gelatin after 4 h. The degradation of neat gelatin in the SIF environment appeared evident with a degradation time of a few minutes. The gelatin and RS gelatin in SGF dissolved in a few minutes (data not shown). Interestingly, once strained, the MO-07/RS composite showed elastic properties without cracks (Figure 5c). Therefore, by modulating gelatin composition by physical blending peptide-modified RS, we could match both the elastic and enteric properties of ingestible gelatin. By the comparison of the FTIR spectra of gelatin [41], MO-07/RS, and MO-07/RS-modified gelatin, the secondary silk protein structure [42] was determined to consist of side chains (1605–1616 cm^−1^), *β*-sheets (1616–1622, 1622–1628, 1628–1638, and 1697–1704 cm^−1^), random coils (1638–1647 and 1647–1656 cm^−1^), *α*-helices (1656–1663 cm^−1^), and turns (1663–1671, 1671–1686, and 1686–1697 cm^−1^). The addition of gelatin to MO-07/RS increased the *β*-sheets content (Figure S7). These results indicate that the interaction between MO-07/RS and gelatin induced more stable structures in the gelatin in terms of elasticity and insolubility.

## 3. Materials and Methods

### 3.1. Materials

Fmoc-Ser(tBu)-Wang resin was from Iris Biotech AG (Marktredwitz, Germany). Peptide grade N,N-dimethylformamide (DMF), activators N,N′-diisopropylcarbodiimide (DIC), Oxyma Pure, all Fmoc-L amino acids, trifluoroacetic acid (TFA), triisopropyl silane (TIS), diisopropyl ether (iPr2O), 2-propanol, and HPLC plus water were purchased from Sigma Aldrich (Milan, Italy). HPLC-grade acetonitrile (ACN) was purchased from Carlo Erba (Milan, Italy). Salts used for preparing PBS buffer (NaCl, KCl, KH_2_PO_4_, and Na_2_HPO_4_) were purchased from Sigma Aldrich (Milan, Italy). EDC*HCl (N-(3-Dimethylaminopropyl)-N′-ethylcarbodiimide hydrochloride and NHS (N-Hydroxysuccinimide) used for the silk functionalisation were from Sigma Aldrich (Milan, Italy). Silk cocoons were supplied from a local farm. Sodium hydrogen carbonate (NaHCO_3_, > 99.5%), calcium chloride (CaCl_2_, anhydrous >93%), formic acid (FA, reagent grade > 95%), gelatin from porcine skin Type A, hydrochloric acid (HCl), and sodium hydroxide (NaOH) were supplied by Merck.

### 3.2. Peptide Synthesis

Peptide MO-07 (YRGDS, reported in Figure S2) was prepared by induction-assisted SPPS in a PurePep^®^ Chorus instrument (Gyros Protein Technologies, Uppsala, Sweden), using a Fmoc-Ser(tBu)-Wang resin (loading: 0.6 mmol/g), with a single coupling protocol, and a 0.25 mmol synthesis scale. The yield of crude peptide was 80.5%.

### 3.3. Peptide Purification

Crude MO-07 was purified on a CombiFlash^®^ NextGen 300+ Teledyne ISCO instrument (Lincoln, NE, USA) with a Teledyne ISCO RediSep^®^ C18 Aq 15.5 g gold column, as reported in Figure S3. Eluents were 0.1% *v*/*v* TFA in milliQ H_2_O (solvent A) and 0.1% *v*/*v* TFA in acetonitrile (solvent B). The purification method started with 3 column volumes (CV) of solvent A to eliminate salts from the product. Then, the solution went from 0% to 20% of B in A for 10 CV, with an isocratic separation at 10.1% of B (because of MO-07 peak elution) for 2 CV (Figure S3). At the end of the separation, the column was washed with 80% B for 2 CV.

### 3.4. Peptide Characterisation and Preparation of the Calibration Curve

Peptide MO-07 was analysed with a UHPLC Thermo Dionex Ultimate 3000 system coupled with MSQ plus single quadrupole ESI-MS (Waltham, MA, USA) using an Acquity column UPLC CSH™ C18 (1.7 μm, 2.1 × 100 mm) (Milford, MA, USA). The final UHPLC purity of the peptide was >98%. UV chromatogram and mass spectrum for the pure peptide MO-07 are reported in Figures S4 and S5, respectively. Peptide MO-07 was weighed with an analytical balance, and precise peptide concentrations in milliQ H_2_O were prepared in volumetric flasks. Peptide concentration of 1 mg/mL, 0.5 mg/mL, 0.2 mg/mL, and 0.1 mg/mL were analyzed with a UHPLC Thermo Dionex Ultimate 3000 instrument with an Acquity column UPLC CSH™ C18 (1.7 μm, 2.1 × 100 mm). The eluent system was 0.1% *v*/*v* TFA in milliQ H_2_O (solvent A) and 0.1% *v*/*v* TFA in acetonitrile (solvent B). The gradient was from 0.1% to 5% of B in A in 5 min, and the acquisition was made at λ: 215 nm. The injection volume used was 10 μL, and the quantification was made by measuring the area under the peak. As reported in Figure S6, the curve R^2^ was 0.9999, and the equation for the curve was: y = 134.21x − 1.2371.

### 3.5. Silk Fibres Functionalisation

*Bombyx mori* silk cocoons (10 g) were boiled (i.e., at 100 °C) for 30 min in 200 mL of water containing 5 g of NaHCO_3_. The extract fibres were washed twice with water and dried at room temperature under a chemical hood in laminar flow. To prepare the degummed silk fibres (hereon DSF) for the functionalisation, 833.8 mg of DSF was weighed, placed in a 50 mL pre-weighed tube, and covered with 30 mL of PBS (10 mM, pH 6.5). After being left there for 30 min, PBS was removed from the well with a dispensable graduated plastic pipet, and the DSF were squeezed with a spatula to remove as much PBS as possible. The -COOH groups were activated by treating the wet DSF with a 40 mL of a freshly prepared solution of 0.5 mM EDC*HCl (N-(3-Dimethylaminopropyl)-N′-ethylcarbodiimide hydrochloride) and 0.7 mM NHS (N-Hydroxysuccinimide) in PBS pH 6.5 for 15 min. After that time, the activation solution was removed from the well with a dispensable graduated plastic pipet. Forty millilitres of a freshly prepared 0.2 mg/mL peptide solution in PBS pH 7.4 was added to the DSF and left in the well for 2.5 h. One millilitre of the so-prepared peptide solution was shelved at “time zero” for further UHPLC-MS acquisitions. The peptide solution, after 2.5 h, was recovered in a 50 mL tube, and the DSF was washed with PBS pH 7.4 (twice, 5 min each). Additional washes were performed with milliQ H_2_O (twice, 5 min each). The DSF was then left in the tube and freeze-dried at the lyophilizer. To quantify, by indirect measurement, the concentration of peptide MO-07 linked to DSF, samples of the peptide solutions were taken before and after the reaction and analysed by UHPLC in the same conditions used to prepare the calibration curve. This procedure enabled us to define the quantity of peptide consumed by the reaction, corresponding to the peptide linked to DSF.

### 3.6. Gelatin and Regenerated Silk Composite Preparation

Neat and peptide functionalised silk fibres (MO-07/DSF) were dissolved in 10 mL of formic acid (FA), containing an amount of CaCl_2_ equal to 60:40 concerning the weight of the dried fibroin fibres (i.e., 0.65 g), at 30 °C for 1 h (i.e., 0.43 g). Films were then fabricated by drop-casting the solutions into Petri dishes with a diameter of 5 cm and left to evaporate at 40 °C for 4 h. Gelatin prepared from porcine skin (Type A) was purchased from Merck. Distilled water (20 mL) was added to porcine skin (2 g), and gelatin was obtained by drop-casting into Petri dishes with a diameter of 5 cm, leaving for 24 h at 6 °C. Regenerated silk (RS) (1.08 g) was then re-dispersed in water (20 mL), and 10 wt % (calculated with respect to the water) of gelatin was added. RS-based gelatin solution was deposited into a Petri dish and left to evaporate at room temperature. Due to the hydrophilic nature of the peptide (see below), peptide-functionalised RS (MO-07/RS) (1.08 g) were then re-dispersed in FA (20 mL), and 10 wt % (calculated with respect to the FA) of gelatin was added. Gelatin-based MO-07/RS solution was deposited into a Petri dish and left to evaporate at room temperature.

### 3.7. Characterizations

The contact angles of DSF and MO-07-modified DSF were measured after placing a water droplet onto the fibres. A water droplet of about 5 µL was applied, and photographs were taken at an interval of 5 s. The contact angles were measured in a goniometer (FTÅ200, First Ten Ångstroms, Inc., Portsmouth, NH, USA) using the FTÅ Drop Shape Analysis Software, Version 2.0 (2002, Portsmouth, NH, USA).

FTIR investigation on MO-07 peptide, RS, and peptide-modified RS films was performed using a PerkinElmer spectrometer in ATR mode. Measurements were performed with a resolution of 4 cm^−1^, and the number of scans was 200 for each spectrum. The background spectra were recorded before each spectrum. The measurements were performed at room temperature. The spectra were collected in the range of 4000 to 400 cm^−1^.

The FTIR spectra of MO-07/RS and MO-07/RS–gelatin composite were deconvoluted by subtracting a linear baseline and applying a Gaussian deconvoluting curve by Origin 9 software (Origin Lab Corporation, Northampton, MA, USA). The spectra were collected in the range 1750 to 1450 cm^−1^, the amide I and amide II bands. Deconvolution was performed using Lorentzian line shape, and the relative content of the structure was obtained by curve-fitting and measuring the relative areas.

The mechanical properties of the RS and MO-07/RS films were tested with a tensile testing machine (Lloyd Instr. LR30K, Bognor Regis UK). Rectangular samples (1.5 cm × 3 cm × 100 μm) were stretched with a strain rate of 5 mm·min^−1^ using a 50 N load cell. Three samples per composition were tested after treatment in climatic chamber for 4 hours at 40 °C and 70% of RH. The compression test on gelatin modified with RS and MO-07/RS was performed on cylinders obtained by dropping the solutions on the mould of cylindrical shape with the dimensions of 10 mm in diameter and 16 mm in length. Two cylindrical metal plates performed the test with a compression rate of 0.1 mm/s.

To simulate gastric fluid conditions (pH, 1.2), 2 g of NaCl and 8.3 mL of concentrated HCl were dissolved and adjusted to 1000 mL with water. Similarly, to simulate intestinal fluid (pH~6.8), 6.8 g of KH_2_PO_4_ and 0.896 g of NaOH were dissolved in water and adjusted to 1000 mL. Degradation was measured as the percent weight loss over time calculated as the difference between the initial and final weight normalised to the initial weight. To perform the dissolution study, gelatin-based RS and MO-07/RS were cut into pieces and submerged in 10 mL of either gastric or intestinal fluid at 37 °C at different times. Gelatin containing MO-07/RS was treated for 60 min. in gastric fluid at 37 °C, clamped between two grips, and strained from 5 mm to 25 mm.

## 4. Conclusions

This study describes a method to prepare peptide-functionalized regenerated silk fibroin and its use to obtain enteric and elastic polymer gelatin, paving a way for a direct application in the fabrication of devices to reach inaccessible regions in the small intestine. The retention of elastic properties in gastric fluid conditions envisions the design of novel devices, including those for ingestible electronics and prolonged drug delivery.

## Data Availability

Not applicable.

## Supplementary Materials

The following supporting information can be downloaded at: https://www.mdpi.com/article/10.3390/molecules27144605/s1. Figure S1: Photographs of the RS gelatin and MO-07/RS gelatin composite during the compression test. Figure S2: Structure of MO-07. Figure S3: Chromatogram of MO-07 after purification. Figure S4: Chromatogram of MO-07 after purification. Figure S5: Mass spectrum of peptide MO-07 after purification. Figure S6: Calibration curve for peptide MO-07. Figure S7: The difference between FTIR spectra for porcine skin gelatin and MO-07/RS gelatin composite.

## Sample Availability

Samples of the compounds are available from the authors.
