# Supplementary material for: Peptide-Functionalized Silk Fibers as a Platform to Stabilize Gelatin for Use in Ingestible Devices
Source: Molecules. 2022 Jul 19;27(14):4605. doi: 10.3390/molecules27144605 (PMC9318617; doi:10.3390/molecules27144605)
Supplement: Supplementary file 1 [file molecules-27-04605-s001.zip › molecules-1819901-supplementary.pdf]

Supporting Information

Peptide-Functionalized Silk Fibers as a Platform to Stabilize Gelatin for Use in Ingestible Devices

Luca Valentini [1,*], Lorenzo Pacini [2], Fosca Errante [3], Cecilia Morchio[4], Beatrice Sanna[4], Paolo Rovero [3], and Antonino Morabito [4]

[1] Civil and Environmental Engineering Department, University of Perugia, Strada di Pentima 4, Terni, 05100, Italy; luca.valentini@unipg.it

[2] Interdepartmental Research Unit of Peptide and Protein Chemistry and Biology, Department of Chemistry "Ugo Schiff", University of Florence, 59100 Sesto Fiorentino, Italy; l.pacini@unifi.it

[3] Interdepartmental Research Unit of Peptide and Protein Chemistry and Biology, Department of NeuroFarBa, University of Florence, 50019 Sesto Fiorentino, Italy; fosca.errante@unifi.it, paolo.rovero@unifi.it

[4] Dipartimento Neuroscienze, Psicologia, Area del Farmaco e della Salute del Bambino NEUROFARBA, Università degli Studi di Firenze, Viale Pieraccini 6, Firenze, 50121, Italy; cecilia.morchio@unifi.it, beatrice.sanna@unifi.it, antonino.morabito@unifi.it

* Correspondence: luca.valentini@unipg.it;

Supporting Information

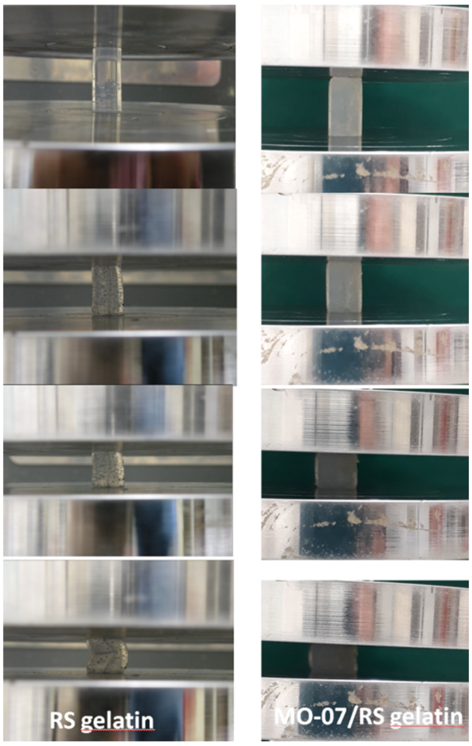

Figure S1. Photographs of the RS gelatin and MO-07/RS gelatin composite during the compression test.

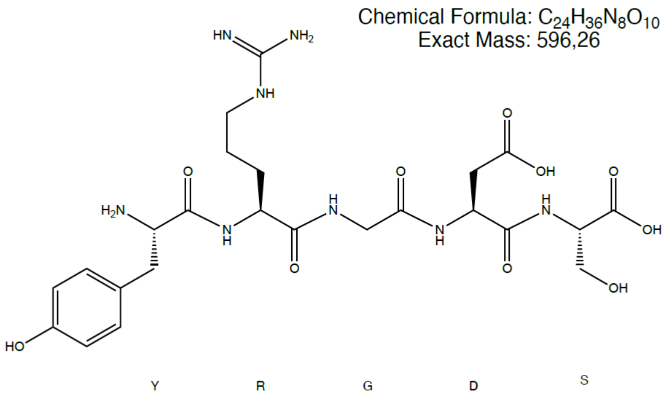

Figure S2. Sequence, chemical formula, and exact mass of peptide MO-07.

Supporting Information

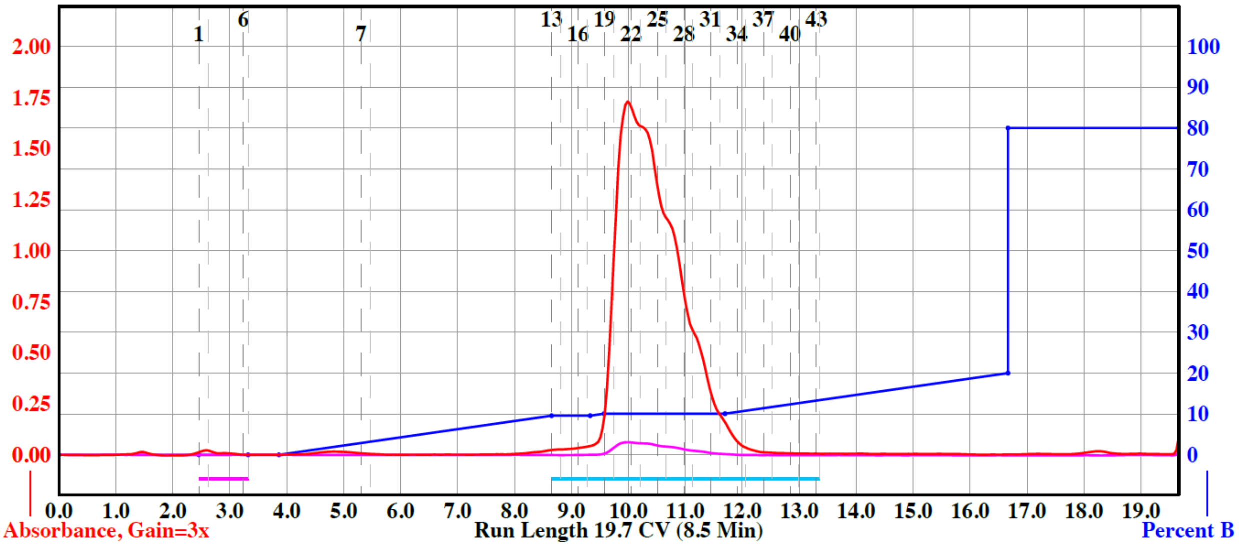

Figure S3. Chromatogram of MO-07 purification. Instrument used: CombiFlash® NextGen 300+ Teledyne ISCO. Column used: Teledyne ISCO RediSep® C18 Aq 15.5 g gold column. Eluents system: solvent A) 0.1% v/v TFA milliQ H2O, solvent B) 0.1% v/v TFA ACN. Gradient used: from 0% to 20% of B and lasts 10 CV.

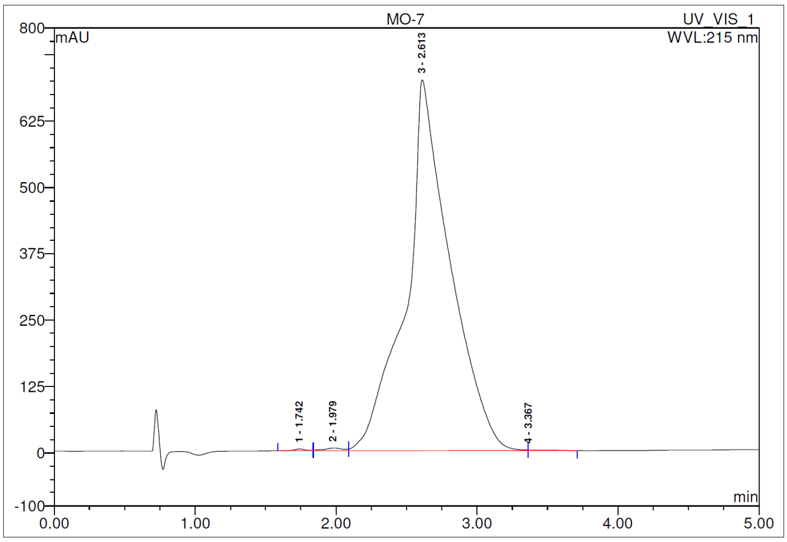

No.	Ret.Time min	Peak Name	Height mAU	Area mAU*min	Rel.Area %	Amount	Type
1	1.74	n.a.	3.397	0.318	0.12	n.a.	BM *
2	1.98	n.a.	4.901	0.777	0.30	n.a.	M *
3	2.61	n.a.	698.004	253.966	99.48	n.a.	M *
4	3.37	n.a.	1.023	0.229	0.09	n.a.	MB*
Total:			707.326	255.290	100.00	0.000	

Figure S4. Chromatogram of MO-07 after purification. Instrument used: UHPLC Thermo Dionex Ultimate 3000. Column used: Acquity column UPLC CSH™ C18 (1.7 µm, 2.1 x 100 mm). Eluents system: solvent A) 0.1% v/v TFA milliQ H2O, solvent B) 0.1% v/v TFA ACN. Gradient used: from 0.1% of ACN to 5% CAN in 5 minutes. Acquisition λ: 215 nm.

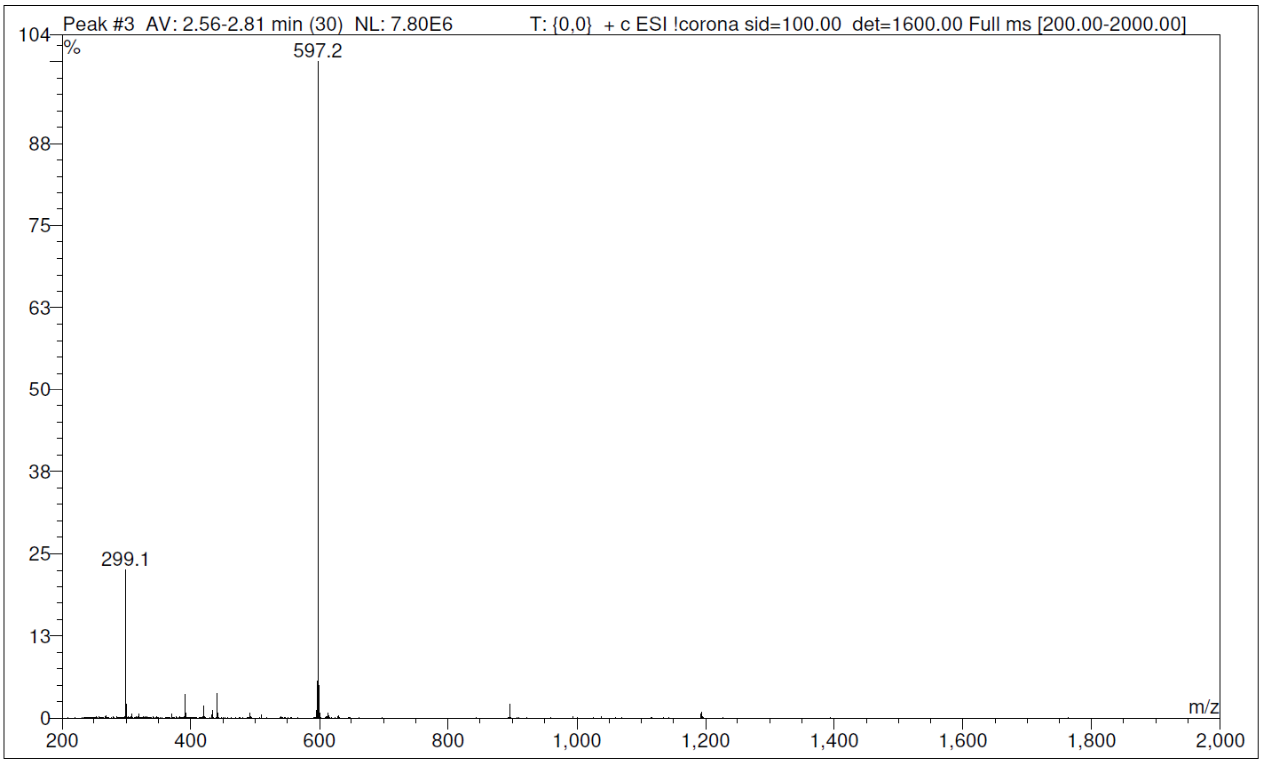

Figure S5. Mass spectrum of peptide MO-07 after purification. Instrument used: MSQ plus single quadrupole ESI mass spectrometer, Thermo Scientific.

Supporting Information

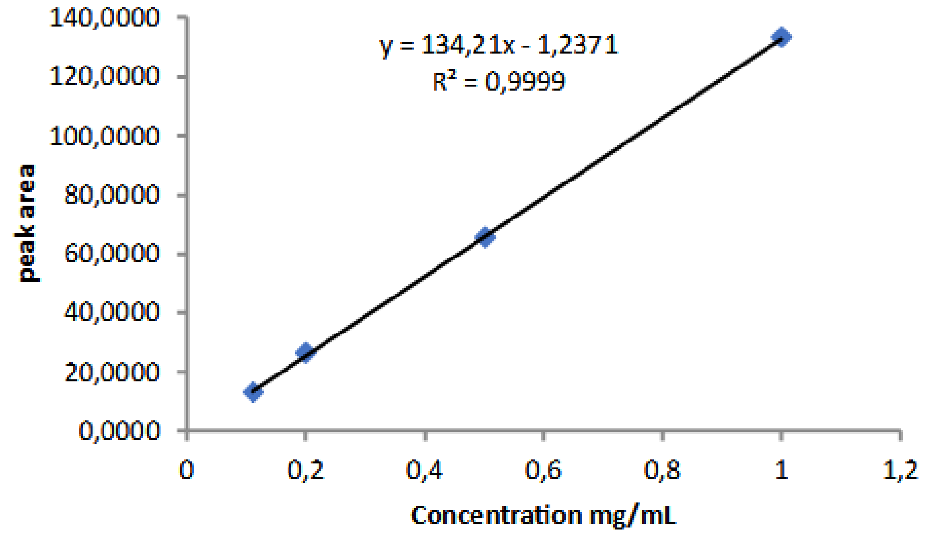

Figure S6. Calibration curve for peptide MO-07.

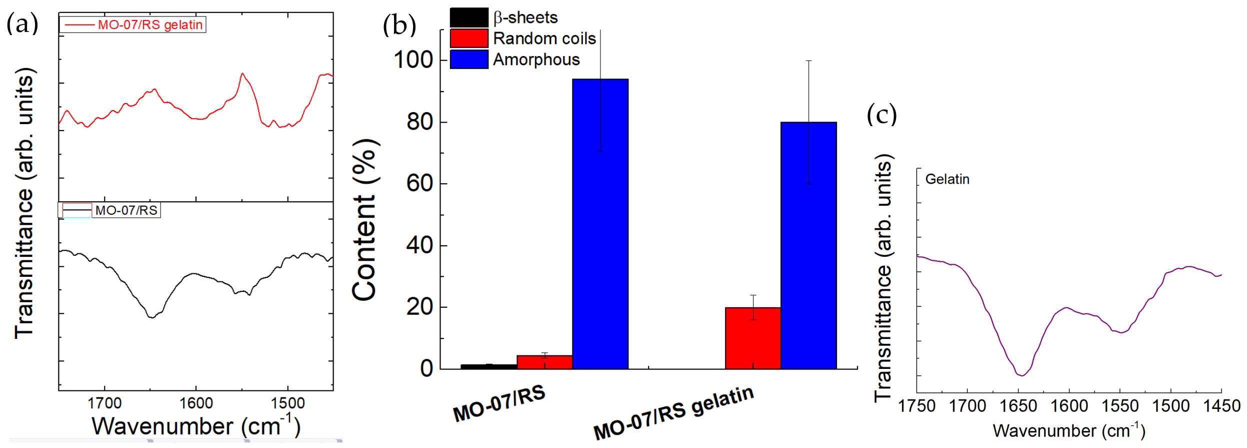

Figure S7. (a) FTIR spectra for MO-07/RS and MO-07/RS gelatin composite with (b) the structural conformation ratios derived from deconvoluted FTIR spectra. (c) FTIR spectrum of neat gelatin.
